# Supplementary material for: Reciprocal discoidin domain receptor signaling strengthens integrin adhesion to connect adjacent tissues
Source: bioRxiv. 2023 May 16:2023.03.14.532639. Originally published 2023 Mar 15. Preprint. [Version 2] doi: 10.1101/2023.03.14.532639 (PMC10055161; doi:10.1101/2023.03.14.532639)
Supplement: Supplement 4 [file NIHPP2023.03.14.532639v2-supplement-4.pdf]

bioRxiv preprint doi: https://doi.org/10.1101/2023.03.14.532639; this version posted May 17, 2023. The copyright holder for this preprint (which was not certified by peer review) is the author/funder, who has granted bioRxiv a license to display the preprint in perpetuity. It is made available under aCC-BY 4.0 International license.

Figure 3—figure supplement 1

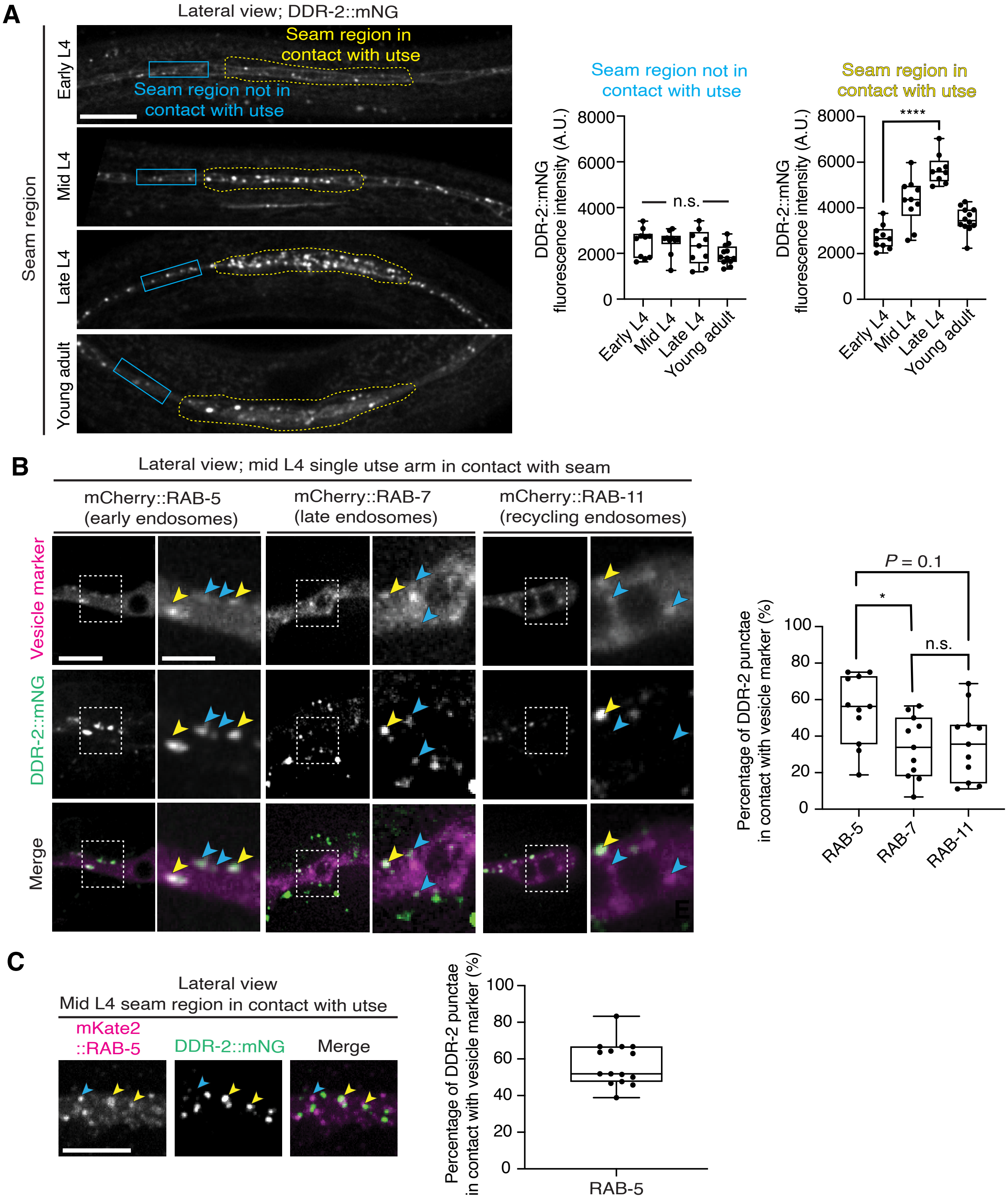

DDR-2 is found within endocytic vesicles.

Related to ***Figure 3***. (**A**) Lateral fluorescence images of DDR-2::mNG in the seam cells from the early L4 to young adult stages. Note that these images are the same animals as in ***Figure 3B*** with a larger cropped field of view to include regions of the seam not in contact with the utse. Quantification of mean fluorescence intensity in the blue and yellow boxed regions is shown on the right ($n \geq 9$ all stages). Boxplots for yellow boxed regions are reproduced from ***Figure 3B***. n.s. (not significant), $P > 0.05$; Kruskal-Wallis H test. ****$P \leq 0.0001$, one-way ANOVA with post hoc Dunnett's test. (**B**) Lateral fluorescence images of DDR-2::mNG within a single arm of the utse tissue at the mid L4 stage, visualized together with mCherry-tagged RAB-5, RAB-7, or RAB-11. Boxed regions are magnified on the right. Yellow arrowheads denote DDR-2 punctae in contact with the respective vesicle marker, while blue arrowheads correspond to DDR-2 puncta that not in contact with vesicle markers. The boxplot shows quantification of percentage overlap of DDR-2 punctae with vesicle markers (n = 11 animals per marker). *$P \leq 0.05$, n.s. (not significant), $P > 0.05$; one-way ANOVA with post hoc Dunnett's T3 test. (**C**) Left: Lateral fluorescence images of DDR-2::mNG and mKate2::RAB-5 in the seam region contacting the utse. Arrowheads denote DDR-2 punctae in contact with (yellow) or not in contact with (blue) mCherry::RAB-5 punctae. Right: Percentage overlap of DDR-2 punctae with RAB-5 (n = 15). Scale bars, 20 μm. Box edges in boxplots represent the 25th and 75th percentiles, the line in the box denotes the median value, and whiskers mark the minimum and maximum values.

bioRxiv preprint doi: https://doi.org/10.1101/2023.03.14.532639; this version posted May 17, 2023. The copyright holder for this preprint (which was not certified by peer review) is the author/funder, who has granted bioRxiv a license to display the preprint in perpetuity. It is made available under aCC-BY 4.0 International license.

Figure 4—figure supplement 1

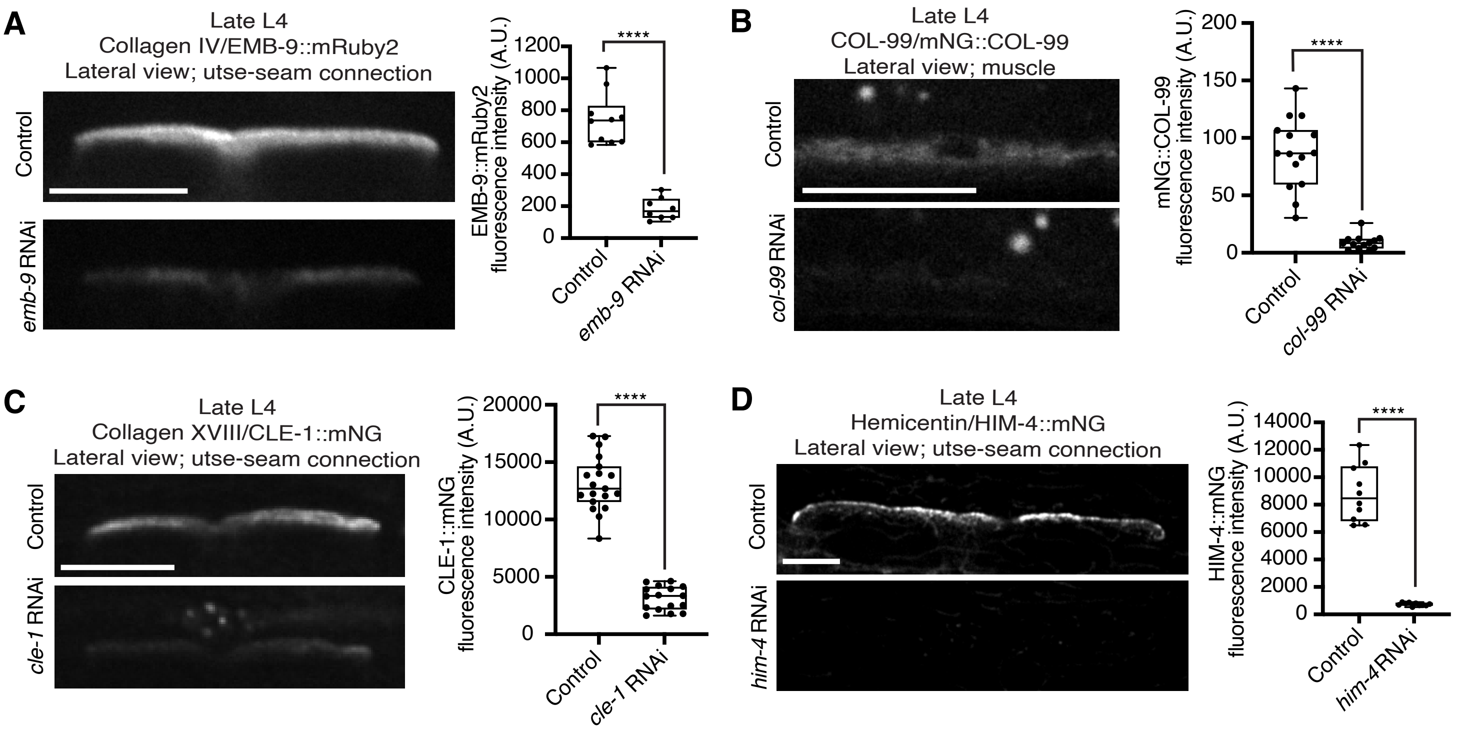

Collagen and hemicentin knockdown efficiencies.
Related to ***Figure 4***. (**A**) Lateral fluorescence images of EMB-9::mRuby2 (type IV collagen) at the utse-seam BM-BM connection in late L4 control or *emb-9* RNAi-treated animals. Mean fluorescence intensity quantified on the right (n ≥ 8 all treatments). (**B**) Lateral fluorescence images of mNG::COL-99 (COL-99) in the body wall muscle tissue in late L4 control or *col-99* RNAi-treated animals. Mean fluorescence intensity quantified on the right (n ≥ 13 all treatments). COL-99 signal in the muscle was analyzed as COL-99 was not detected at the utse-seam connection. (**C**) Lateral fluorescence images of CLE-1::mNG (type XVIII collagen) at the utse-seam BM-BM attachment in late L4 control or *cle-1* RNAi-treated animals. Mean fluorescence intensity quantified on the right (n ≥ 16 all treatments). (**D**) Lateral fluorescence images of HIM-4::mNG (hemicentin) at the utse-seam BM-BM connection in late L4 control or *him-4* RNAi-treated animals. Mean fluorescence intensity quantified on the right (n = 10 all treatments). ****$P \leq 0.0001$, unpaired two-tailed Student's *t* test (EMB-9, CLE-1, and HIM-4) or Mann-Whitney *U* test (COL-99). Scale bars, 20 μm. Box edges in boxplots represent the 25th and 75th percentiles, the line in the box denotes the median value, and whiskers mark the minimum and maximum values.

bioRxiv preprint doi: https://doi.org/10.1101/2023.03.14.532639; this version posted May 17, 2023. The copyright holder for this preprint (which was not certified by peer review) is the author/funder, who has granted bioRxiv a license to display the preprint in perpetuity. It is made available under aCC-BY 4.0 International license.

Figure 5—figure supplement 1

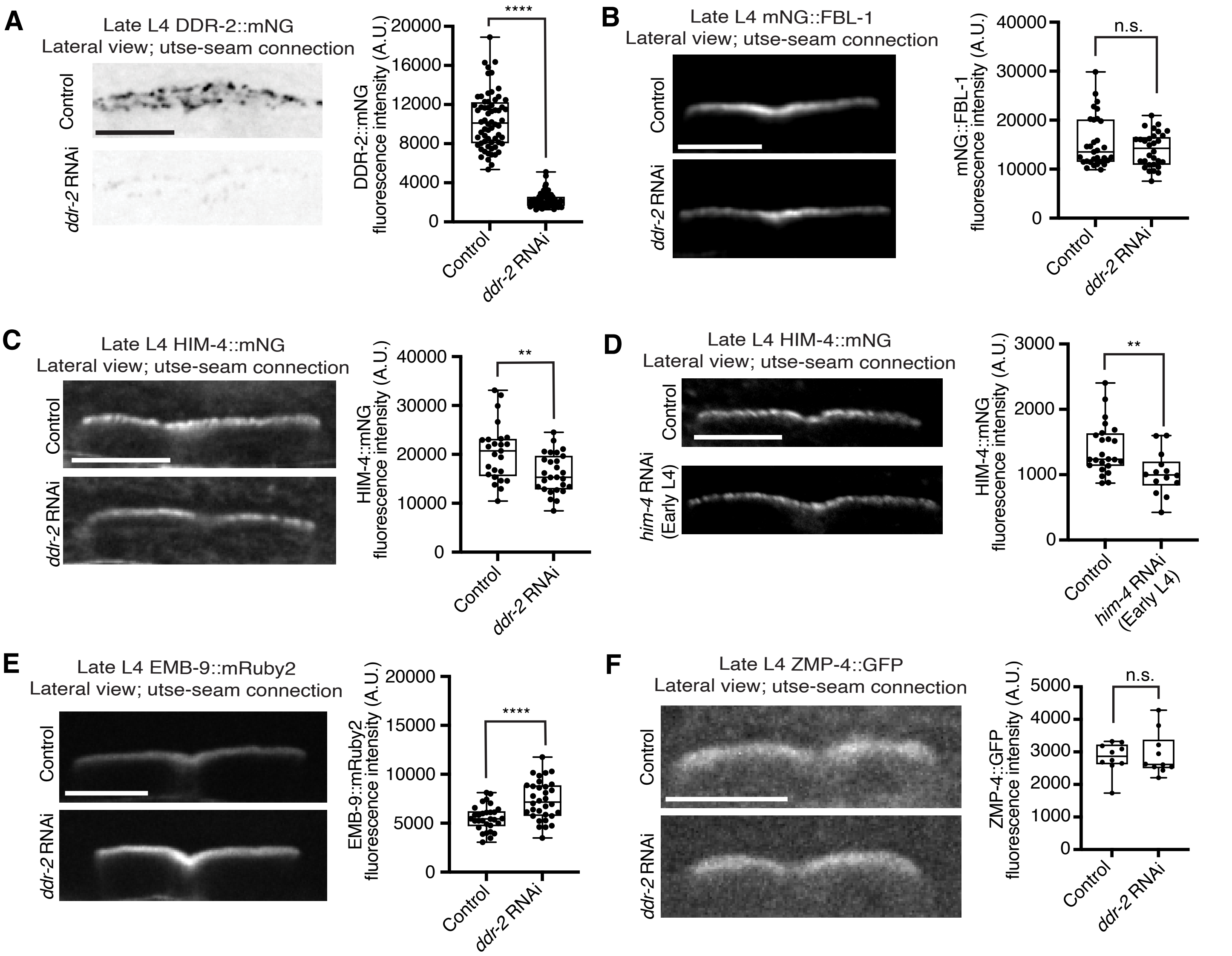

ddr-2 **loss does not reduce functional levels of fibulin, hemicentin, type IV collagen, and matrix metalloproteinase ZMP-4 at the utse-seam connection.** Related to ***Figure 5***. (**A**) Lateral fluorescence images of DDR-2::mNG at the utse-seam BM-BM connection in late L4 control or *ddr-2* RNAi-treated animals. Mean fluorescence intensity quantified on the right (n ≥ 60 all treatments). ****$P \leq 0.0001$, Mann-Whitney *U* test. (**B**) Lateral fluorescence images of mNG::FBL-1 (fibulin) at the utse-seam connection in late L4 control or *ddr-2* RNAi-treated animals. Mean fluorescence intensity quantified on the right (n ≥ 30 all treatments). n.s. (not significant), $P > 0.05$, Mann-Whitney *U* test. (**C**) Lateral fluorescence images of HIM-4::mNG (hemicentin) at the utse-seam tissue linkage in late L4 control or *ddr-2* RNAi-treated animals. Mean fluorescence intensity quantified on the right (n ≥ 25 all treatments). **$P \leq 0.01$, unpaired two-tailed Student's *t* test. (**D**) Lateral fluorescence images of HIM-4::mNG (hemicentin) at the utse-seam connection in late L4 control or *him-4* RNAi-treated animals. RNAi was initiated at the early L4 stage. Mean fluorescence intensity quantified on the right (n ≥ 14 all treatments). **$P \leq 0.01$, unpaired two-tailed Student's *t* test.
(**E**) Lateral fluorescence images of EMB-9::mRuby2 (type IV collagen) at the utse-seam attachment site in late L4 control or *ddr-2* RNAi-treated animals. Mean fluorescence intensity quantified on the right (n ≥ 30 all treatments). ****$P \leq 0.0001$, unpaired two-tailed Student's *t* test. (**F**) Lateral fluorescence images of matrix metalloproteinase ZMP-4::GFP at the utse-seam tissue connection in late L4 control or *ddr-2* RNAi-treated animals. Mean fluorescence intensity quantified on the right (n = 10 all treatments). n.s. (not significant), $P > 0.05$, Mann-Whitney *U* test. Scale bars, 20 μm. Box edges in boxplots represent the 25th and 75th percentiles, the line in the box denotes the median value, and whiskers mark the minimum and maximum values.

bioRxiv preprint doi: https://doi.org/10.1101/2023.03.14.532639; this version posted May 17, 2023. The copyright holder for this preprint (which was not certified by peer review) is the author/funder, who has granted bioRxiv a license to display the preprint in perpetuity. It is made available under aCC-BY 4.0 International license.

Figure 5—figure supplement 2

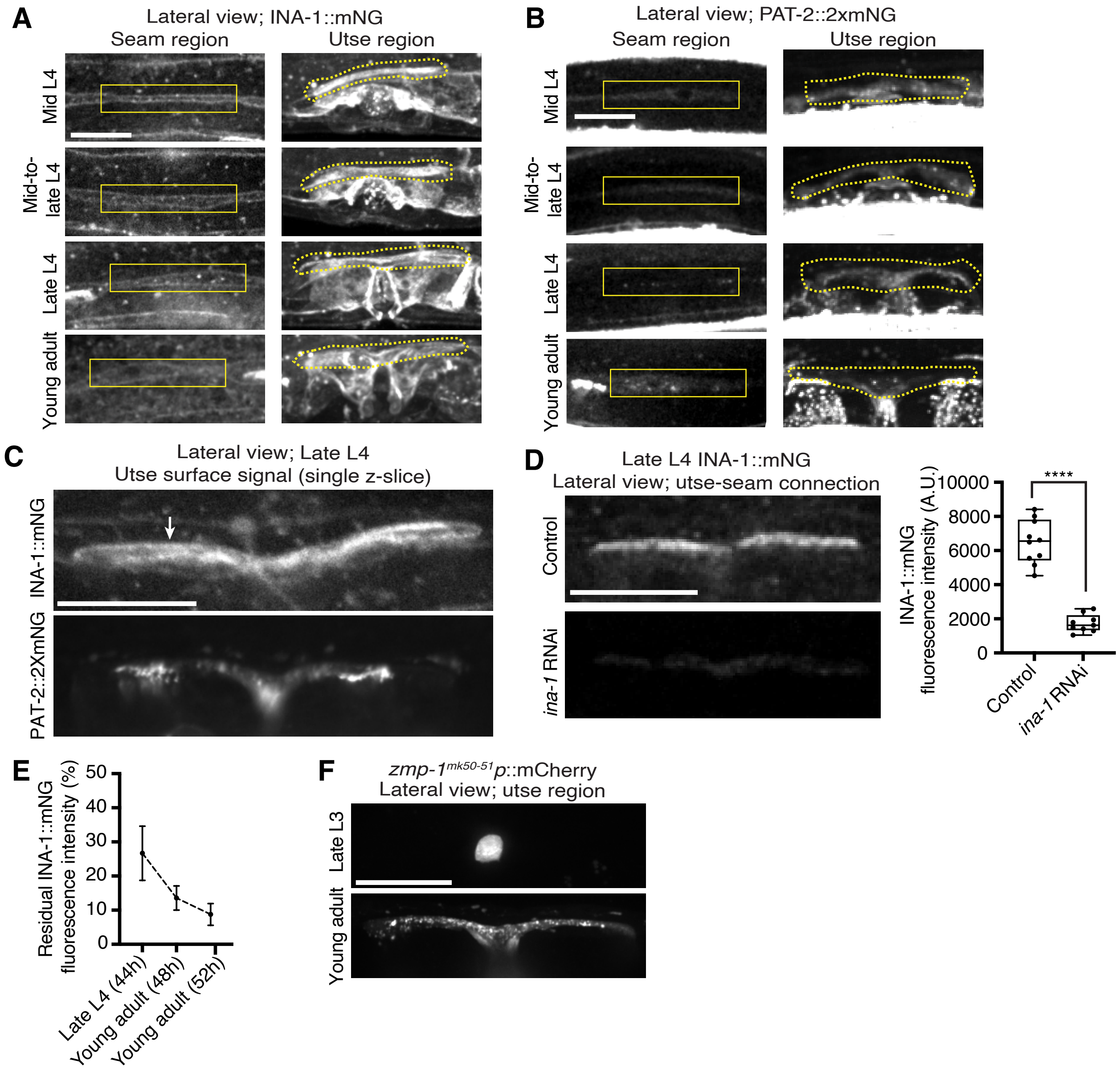

The integrin α subunits INA-1 and PAT-2 are localized within both the utse and seam cells.
Related to ***Figure 5***. (**A**) Lateral fluorescence z-projections of the integrin α subunit INA-1::mNG between the mid L4 and young adult stages. Localization in the seam and utse are indicated by solid and dotted boxed regions respectively (n ≥ 10 animals examined all stages). (**B**) Lateral fluorescence z-projections of the integrin α subunit PAT-2::2XmNG between the mid L4 and young adult stages. Localization in the seam and utse are indicated by solid and dotted boxed regions respectively (n ≥ 7 animals examined all stages). (**C**) Lateral fluorescence images of INA-1::mNG and PAT-2::2XmNG at the z-plane of the utse surface in late L4 animals (n ≥ 10 animals examined each). Arrow indicates strong INA-1 signal at the utse surface. (**D**) Lateral fluorescence images of INA-1::mNG at the utse-seam connection in late L4 control or *ina-1* RNAi-treated animals. Mean fluorescence intensity quantified on the right (n ≥ 9 all treatments). *****P* ≤ 0.0001, unpaired two-tailed Student's *t* test. (**E**) Quantification of residual INA-1::mNG fluorescence intensity at the utse-seam interface at the indicated developmental stages following *ina-1* knockdown initiated at the L1 stage. Data are represented as percentages of mean INA-1::mNG at the respective stages in control animals (n ≥ 7 animals examined all stages). Error bars denote SD. (**F**) Lateral fluorescence z-projections of mCherry driven by the *zmp-1*$^{mK50-51}$ promoter in the utse region in late L3 and young adult animals (n = 5 animals examined at each stage).

bioRxiv preprint doi: https://doi.org/10.1101/2023.03.14.532639; this version posted May 17, 2023. The copyright holder for this preprint (which was not certified by peer review) is the author/funder, who has granted bioRxiv a license to display the preprint in perpetuity. It is made available under aCC-BY 4.0 International license.

Figure 6—figure supplement 1

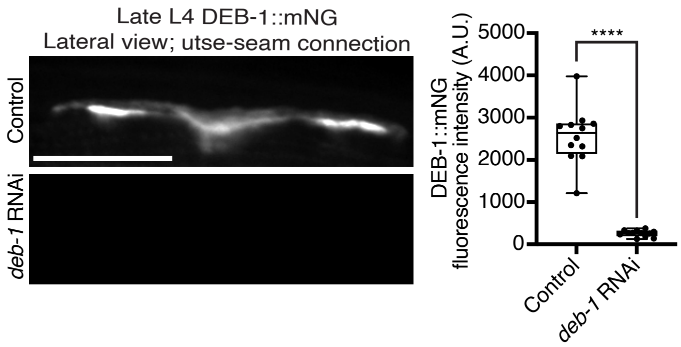

***deb-1* knockdown efficiency.**
Related to ***Figure 6***. Lateral fluorescence images of DEB-1::mNG (vinculin) at the utse-seam connection in late L4 control or *deb-1* RNAi-treated animals. Mean fluorescence intensity quantified on the right (n = 12 all treatments). ****$P \leq 0.0001$, unpaired two-tailed Student's *t* test. Scale bars, 20 μm. Box edges in boxplots represent the 25th and 75th percentiles, the line in the box denotes the median value, and whiskers mark the minimum and maximum values.

bioRxiv preprint doi: https://doi.org/10.1101/2023.03.14.532639; this version posted May 17, 2023. The copyright holder for this preprint (which was not certified by peer review) is the author/funder, who has granted bioRxiv a license to display the preprint in perpetuity. It is made available under aCC-BY 4.0 International license.

Figure 7—figure supplement 1

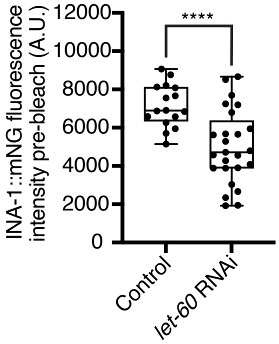

Ras/*let-60* knockdown reduces INA-1::mNG levels at the utse-seam connection.
Related to ***Figure 7***. Quantification of mean INA-1::mNG fluorescence intensity at the utse-seam connection prior to photobleaching in control or Ras/*let-60* RNAi-treated late L4 animals (see representative images in ***Figure 7B***, pre-bleach panel. Control n = 16; *let-60* RNAi n = 25. ****$P \leq 0.0001$, unpaired two-tailed Student's *t* test. Scale bars, 20 μm. Box edges in boxplots represent the 25th and 75th percentiles, the line in the box denotes the median value, and whiskers mark the minimum and maximum values.
